# A comprehensive analysis of the role of native and modified HDL in ER stress in primary macrophages

**DOI:** 10.3389/fcvm.2024.1448607

**Published:** 2024-09-12

**Authors:** Jordan M. Bobek, Gage M. Stuttgen, Daisy Sahoo

**Affiliations:** ^1^ Department of Biochemistry, Medical College of Wisconsin, Milwaukee, WI, United States; ^2^ Cardiovascular Center, Medical College of Wisconsin, Milwaukee, WI, United States; ^3^ Division of Endocrinology & Molecular Medicine, Department of Medicine, Medical College of Wisconsin, Milwaukee, WI, United States

**Keywords:** HDL, modified HDL, oxLDL, primary macrophages, endoplasmic reticulum stress, thapsigargin, lipoprotein, atherosclerosis

## Abstract

**Introduction:**

Recent findings demonstrate that high density lipoprotein (HDL) function rather than HDL-cholesterol levels themselves may be a better indicator of cardiovascular disease risk. One mechanism by which HDL can become dysfunctional is through oxidative modification by reactive aldehydes. Previous studies from our group demonstrated that HDL modified by reactive aldehydes alters select cardioprotective functions of HDL in macrophages. To identify mechanisms by which dysfunctional HDL contributes to atherosclerosis progression, we designed experiments to test the hypothesis that HDL modified by reactive aldehydes triggers endoplasmic reticulum (ER) stress in primary murine macrophages.

**Methods and results:**

Peritoneal macrophages were harvested from wild-type C57BL/6J mice and treated with thapsigargin, oxLDL, and/or HDL for up to 48 hours. Immunoblot analysis and semi-quantitative PCR were used to measure expression of BiP, p-eIF2α, ATF6, and XBP1 to assess activation of the unfolded protein response (UPR). Through an extensive set of comprehensive experiments, and contrary to some published studies, our findings led us to three novel discoveries in primary murine macrophages: (i) oxLDL alone was unable to induce ER stress; (ii) co-incubation with oxLDL or HDL in the presence of thapsigargin had an additive effect in which expression of ER stress markers were significantly increased and prolonged as compared to cells treated with thapsigargin alone; and (iii) HDL, in the presence or absence of reactive aldehydes, was unable blunt the ER stress induced by thapsigargin in the presence or absence of oxLDL.

**Conclusions:**

Our systematic approach to assess the role of native and modified HDL in mediating primary macrophage ER stress led to the discovery that lipoproteins on their own require the presence of thapsigargin to synergistically increase expression of ER stress markers. We further demonstrated that HDL, in the presence or absence of reactive aldehydes, was unable to blunt the ER stress induced by thapsigargin in the presence or absence of oxLDL. Together, our findings suggest the need for more detailed investigations to better understand the role of native and modified lipoproteins in mediating ER stress pathways.

## 1 Introduction

Cardiovascular disease (CVD) remains the leading cause of death globally. Atherosclerosis, one of the major causes of CVD (1), is an inflammatory disease defined by the progressive buildup of plaques consisting of lipid-laden macrophages, cellular debris, cholesterol, lipids, and lipoproteins in the arteries that increases the likelihood of myocardial infarction and stroke (2). Low-density lipoprotein (LDL) and high-density lipoprotein (HDL) play critical roles in the pathogenesis of atherosclerosis due to their roles in transporting cholesterol between tissues and cell types, including macrophages (3).

Macrophages play an important role in the development of atherosclerosis as they regulate inflammatory responses and promote plaque formation (4). The expression of scavenger receptor CD36 on the macrophage cell surface facilitates the uptake of oxidized LDL (oxLDL). The accumulation of esterified cholesterol from oxLDL in the macrophage initiates foam cell formation in the arterial walls that eventually progresses to atherosclerotic plaque formation [reviewed in (5–7)].

A major cardio-protective function of HDL is its role in reverse cholesterol transport (RCT) where it functions to extract cholesterol from lipid-laden macrophages in peripheral tissues and deliver it to the liver for total body cholesterol excretion (8, 9). This process, combined with other anti-atherogenic properties of HDL [reviewed in (10, 11)], helps to reduce or prevent the formation of atherosclerotic plaque. Epidemiological studies have shown that high levels of HDL-cholesterol are correlated with decreased risk for CVD (12). However the failure of HDL-cholesterol raising therapeutics to show clinical benefit (13, 14) suggests that assessing HDL function may be a more relevant indicator of CVD risk than HDL-cholesterol levels themselves (15).

Under conditions of oxidative stress or inflammation, HDL can be susceptible to oxidation and become dysfunctional (16, 17). HDL dysfunction has been studied in many diseases such as coronary artery disease (18) and chronic kidney disease (19), where HDL isolated from these patients had a decreased ability to perform its normal anti-inflammatory and antioxidant functions. Dysfunctional HDL has also been described in diabetes, obesity, and metabolic syndrome (20–22). While the processes that lead to HDL dysfunction are not fully understood, one possible mechanism is by oxidative modification by reactive aldehydes that have been found to alter lipoprotein function in circulation in disease states such as atherosclerosis (23–26). Previous studies from our group have demonstrated that, unlike native HDL, incubation of macrophages with HDL modified by reactive aldehydes inhibits cell migration, promotes lipid accumulation, and increases reactive oxygen species generation (27, 28), all critical steps in initiating the atherosclerotic process. A better understanding of additional pathways by which dysfunctional HDL promotes pathways that lead to atherosclerosis is critical to preventing this disease.

Numerous studies have linked CVD to endoplasmic reticulum (ER) stress (29–38). Protein misfolding and ER stress can be induced by various factors such as hypoxia, calcium deprivation, impaired glycosylation, oxidative stress, and other pathological conditions (39, 40). These events can activate the unfolded protein response (UPR) which, in turn, stimulates signaling pathways to help eliminate ER stress to maintain homeostasis. Three UPR pathways are activated under conditions of ER stress: PERK, IRE1, and ATF6 [reviewed in (41)]. Each branch of the UPR possesses a unique signal transduction mechanism, but all branches work in parallel to upregulate genes encoding ER chaperones and proteins associated with protein degradation and downregulation of protein synthesis (41). Conditions such as inflammation associated with CVD impose high demands on protein-folding machinery in the ER, thereby inducing ER stress (35, 42). On the other hand, ER stress can exert negative effects on CVD progression as it can induce states of inflammation and oxidative stress (30, 34). It has also been demonstrated that ER stress regulates lipid metabolism in macrophages by modulating the expression of membrane cholesterol transporters, specifically, by increasing expression of the oxLDL receptor, CD36 (43), decreasing expression of cholesterol efflux receptors (44), and increasing *de novo* cholesterol synthesis (45, 46), thus resulting in increased lipid accumulation and foam cell generation [reviewed in (47)]. Additionally, expression of the UPR marker, phosphorylated IRE1*α*, was observed in atherosclerotic lesions which also contained high levels of 4-hydroxynonenal (HNE) adducts, a reactive aldehyde produced from lipid peroxidation (48, 49).

Studies examining the role of lipoproteins in mediating ER stress responses remain limited. OxLDL has been shown to upregulate expression of various UPR markers in vascular cells and macrophages, thereby inducing ER stress (49–54). On the other hand, HDL was shown to blunt oxLDL-induced ER stress in the same cell types (55, 56). Based on these findings, the initial goal of this study was to investigate whether modification of HDL by reactive aldehydes would impair HDL's ability to blunt the ER stress response in macrophages. Interestingly, through an extensive set of comprehensive experiments, and contrary to several published studies, we demonstrate that physiological levels of oxLDL do not induce ER stress in murine peritoneal macrophages. Furthermore, ER stress was only induced when oxLDL was co-incubated with the ER stress inducer, thapsigargin. Additionally, we found that the addition of HDL or modified forms of HDL did not protect from ER stress induced by thapsigargin in the presence or absence of oxLDL. Rather both native and modified forms of HDL had similar effects on ER stress as oxLDL.

## 2 Materials and methods

### 2.1 Materials

Ammonium-Chloride-Potassium (ACK) lysis buffer, DNase I, high-capacity cDNA reverse transcriptase kit, Roswell Park Memorial Institute (RPMI) 1640, TaKaRa Ex Taq, thapsigargin (TG), thioglycolate medium, and tunicamycin (TM) were purchased from Thermo Fisher Scientific (Waltham, MA). Human HDL, phosphatase inhibitor cocktail, Sandoz 58-035 (ACAT inhibitor) and 1,1,3,3-Tetramethoxypropane for malondialdehyde (MDA) generation were purchased from Sigma-Aldrich (St. Louis, MO). 4-hydroxynonenal was from Cayman Chemical (Ann Arbor, MI) and acrolein was from Ultra Scientific (North Kingstown, RI). The Total RNA Purification Kit was purchased from Norgen Biotek Corp (Thorold, ON, Canada). Rabbit monoclonal antibody against eIF2α (1:1,000), phospho-eIF2α (1:600) and BiP (1:1,000) were from Cell Signaling (Danvers, MA). Human acetylated low-density lipoprotein, mouse monoclonal antibodies against ATF6 (1:1,000) and beta-Actin (1:2,500), and ProLong Gold Antifade Mountant with DNA stain DAPI, were purchased from Thermo Fisher Scientific (Waltham, MA). [1,2-3H (N)]-Cholesterol was purchased from PerkinElmer (Shelton, CT). Human oxLDL was from Lee Biosolutions, Inc (Maryland Heights, MO). Horseradish peroxidase (HRP)-conjugated donkey-anti-rabbit-IgG (1:10,000) and sheep-anti-mouse IgG secondary (1:10,000) antibodies were purchased from Cytiva (Amersham, UK). All other reagents were of analytical grade.

### 2.2 Animal care

All experimental procedures were approved by the Institutional Animal Care and Use Committee of the Medical College of Wisconsin. Wild type C57BL6/J mice [originally purchased from Jackson Laboratories (Bar Harbor, ME)] were housed and bred in a pathogen-free barrier facility in accordance with federal and institutional guidelines under a normal light-dark cycle and fed a standard chow diet.

### 2.3 Isolation and maintenance of peritoneal murine macrophages

Male and female wild type C57BL6/J mice between 8 and 12 weeks of age received intraperitoneal injections of 4% thioglycolate medium (2 ml per mouse). The inflammatory response was allowed to proceed for 4 days prior to euthanasia by CO_2_ inhalation followed by cervical dislocation. Cold RPMI media containing 10% FBS + 1% Pen/Strep was injected into the peritoneal cavity and the abdomen was gently massaged before the peritoneal fluid was extracted. The peritoneal exudate cells (PECs) were pelleted by centrifugation at 400 × g for 10 min at 4°C. The PECs were resuspended with ACK lysis buffer and incubated on ice for 10 min to lyse contaminating red blood cells. PECs were pelleted again by centrifugation and resuspended in RPMI media containing 10% FBS + 1% Pen/Strep. Macrophages were counted using an automated cell counter (BioRad) and plated for experiments. Cells were maintained in RPMI media containing 10% FBS + 1% Pen/Strep and incubated at 37°C/5% CO_2_ for at least 24 h prior to treatment. The medium was replaced with serum-free RPMI media during treatments for up to 48 h.

### 2.4 Cell Lysis

Whole cell lysates were harvested from cells by incubating with radioimmunoprecipitation assay (RIPA) buffer containing protease inhibitors (aprotinin, leupeptin, phenylmethylsulfonylfluoride and pepstatin) and phosphatase inhibitor cocktail on ice for 10 min. Lysates were clarified by centrifugation at 8,000 × g for 10 min at 4°C. Total protein concentration of lysates was determined by the Lowry method (57).

### 2.5 Isolation and modification of human lipoproteins

Human LDL (d = 1.063 g/ml) and HDL (d = 1.210 g/ml) were isolated from human plasma via density gradient ultracentrifugation (58). Lipoproteins were dialyzed overnight against PBS at 4°C and purity was verified using fast protein liquid chromatography (FPLC) analysis. To generate oxLDL, isolated LDL was oxidized by dialysis against 5 µM copper sulfate (CuSO_4_) in PBS for 6 h at 37°C, and the reaction was stopped by dialysis in PBS containing 0.02% (0.54 nM) EDTA overnight at 4°C. An additional dialysis against PBS for 6 h at 4°C was performed to remove any remaining EDTA (59). Thiobarbituric acid reactive substance (TBAR) and electrophoretic mobility shift (EMSA) assays were performed to verify oxidation levels and changes in charge, respectively. Lipoproteins were used within three weeks of isolation and stored under argon in 4°C to prevent oxidation. To generate the modified forms of HDL, HDL was incubated with 500 µM HNE, 250 µM acrolein, or 15 µM MDA at 37°C for 18 h as previously described (28). MDA was generated prior to each reaction (60). Each reaction was terminated with 20-fold molar excess of aminoguanidine hydrochloride to scavenge excess aldehyde. Modified forms of HDL were used in experimentation immediately and modification was verified using EMSA assays and immunoblot analyses as previously described (28).

### 2.6 Immunoblot analysis

Cellular lysates (25 µg) combined with an equal volume of sample treatment buffer containing 5% 2-mercaptoethanol were boiled for 5 min at 95°C, separated by 10% SDS-PAGE, and wet-transferred to a nitrocellulose membrane. The membranes were blocked in 5% milk or BSA in tris-buffered saline with tween (TBST) at room temperature and then incubated with the primary antibody in 1% milk or BSA overnight at 4°C. Membranes were washed 3 times in TBST at room temperature and then incubated with horseradish peroxidase-conjugated secondary antibody in 1% milk at room temperature for 1 h. SuperSignal West Pico or Fempto Chemiluminescent Substrate (Thermofisher) was allowed to react with membranes for 5 min before blots were imaged using a ChemiDoc MP. The intensity/optical density values of the bands were measured using Bio Rad Image Lab Software and normalized by a house-keeping protein (β-actin).

### 2.7 RNA extraction and semi-quantitative reverse transcriptase PCR analysis

Total RNA was extracted using the Total RNA Purification Kit (Norgen Biotek Corp.) following the manufacturer's instructions. Contaminating DNA was digested with DNase I (ThermoFisher) before 1 µg total RNA was reverse-transcribed into single-stranded cDNA using the high-capacity cDNA reverse transcriptase kit (ThermoFisher). The following primers were designed and purchased from IDT Technologies (Coralville, IA): XBP1 forward primer: 5’-AGA GGT GGA GGC CAA GGG GAG T-3’, reverse primer: 5’-GGG TCC AAC TTG TCC AGA ATG CCC-3’ (length of 200 bp for unspliced product and 174 bp for spliced product). The primers were synthesized by TaKaRa Ex Taq (Fisher) and the products were separated on a 3% agarose gel containing ethidium bromide and imaged using a ChemiDoc MP.

### 2.8 Free cholesterol efflux to HDL

Bone-marrow derived macrophages were plated at a density of 1.0 × 10^6^ cells in a 12-well plate and incubated at 37°C/5% CO_2_ overnight. Cells were labeled with [^3^H]cholesterol for 24 h and then allowed to equilibrate for 24 h in RMPI containing 0.2% BSA before adding 50 μg/ml HDL for 4 h. Free cholesterol efflux to HDL was calculated by dividing the radioactive counts in the media by the total radioactivity in the media and cells. All incubations were performed in the presence of ACAT inhibitor.

### 2.9 Macrophage foam cell formation

Peritoneal macrophages were seeded in a 6-well plate on a cover slip at a density of 0.75 × 10^6^ cells per well and incubated at 37°C/5% CO_2_ overnight. Media was replaced with serum-free RMPI containing 0–25 μg/ml oxLDL or acetylated LDL (acLDL) for 24 h at 37°C/5% CO_2_. The cells were washed, fixed in 4% paraformaldehyde, and then treated with a 0.156% Oil Red O (ORO) stain for 5 min. After washing the cells, the cover slips were mounted onto a glass slide with ProLong Gold Antifade Mountant with DAPI and sealed with clear nail polish. The slides were imaged using a Keyence All-in-One Fluorescence Microscope (BZ-X800) and ImageJ was used to quantify the total number of “foam cells” and total number of cells stained with DAPI.

### 2.10 Electrophoretic mobility shift assay

Lipoproteins (10 μg) were separated on a 1% agarose gel for 30 min at 100 V. Gels were stained with Coomassie Blue for 30 min and de-stained overnight. The charge differences between the native and oxidized forms of lipoproteins were calculated by comparing migration distances on the gel.

### 2.11 Data normalization and statistical analyses

Each experiment was repeated at least three times, with macrophages harvested from three independent isolations. Densitometry data was normalized to the housekeeping gene and expressed as either relative intensity or further normalized to the positive control to serve as the baseline. Statistical analyses were performed using GraphPad Prism (San Diego, CA), with details of each analysis provided in Figure Legends.

## 3 Results

### 3.1 Thapsigargin induces ER stress in murine peritoneal macrophages

**Figure 1:**
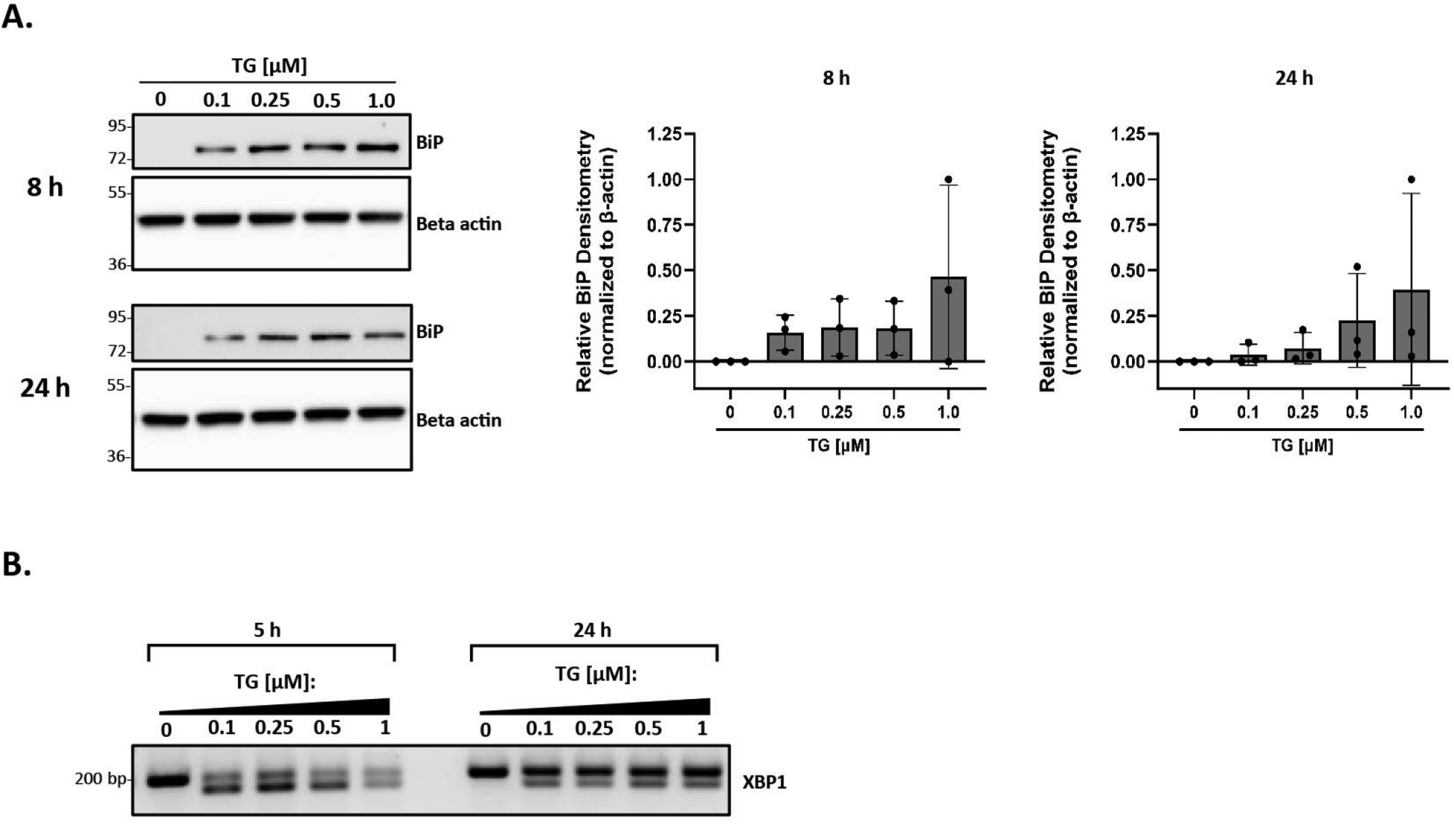
Thapsigargin induces ER stress in murine peritoneal macrophages. Murine peritoneal macrophages were treated with 0–1 µM thapsigargin (TG) (in a final concentration of 0.5% DMSO) for 8 h **(A)** or 24 h **(B)** Whole cell lysates were harvested and BiP expression was analyzed by immunoblot analyses. Densitometry was used to calculate changes in band intensity normalized to the housekeeping gene, beta-actin. Blots shown are representative of *n* = 3 independent macrophage isolations. **(C)** RNA was isolated and reverse transcribed to cDNA. Primers designed for the unspliced (200 bp) and spliced (174 bp) forms of XBP-1 were used in a semi-quantitative PCR reaction and products were separated on a 3% agarose gel. The gel shown is representative of *n* = 3 independent macrophage isolations.

We applied a highly systematic approach to assess the role of lipoproteins in mediating ER stress. A key first step in this process was to establish a good positive control for ER stress in our primary peritoneal macrophage model system. Thapsigargin is a known inducer of ER stress due to its role in inhibiting the sarco-/endoplasmic reticulum calcium ATPase and disrupting calcium homeostasis (61). While it is a commonly-used positive control in ER stress experiments, to our knowledge, there are no studies that address appropriate concentration ranges of thapsigargin for use in primary macrophages. We treated peritoneal macrophages from C57BL/6 wild-type mice with increasing concentrations of thapsigargin ranging from 0.1 to 1 µM for 8 and 24 h and analyzed BiP expression. BiP, also referred to as GRP78, is a central regulator for ER stress and can control the activation of the three ER stress pathways [reviewed in (62)]. While all tested concentrations of thapsigargin induced BiP expression, the lowest concentration of 0.1 µM was sufficient to induce BiP expression at both 8 and 24 h (Figures 1A,B). Additionally, we examined the splicing of XBP-1 at the mRNA level to ensure the IRE1 UPR pathway responded in a similar fashion. Comparable findings were observed in which all concentrations of thapsigargin induced splicing of XBP-1, with 0.1 µM thapsigargin being sufficient at both time points to induce splicing (Figure 1C). We therefore decided to use 0.1 µM thapsigargin (unless otherwise specified) as a positive control for ER stress in all experiments described in this study.

### 3.2 OxLDL alone does not induce ER stress in murine peritoneal macrophages at physiological concentrations

**Figure 2:**
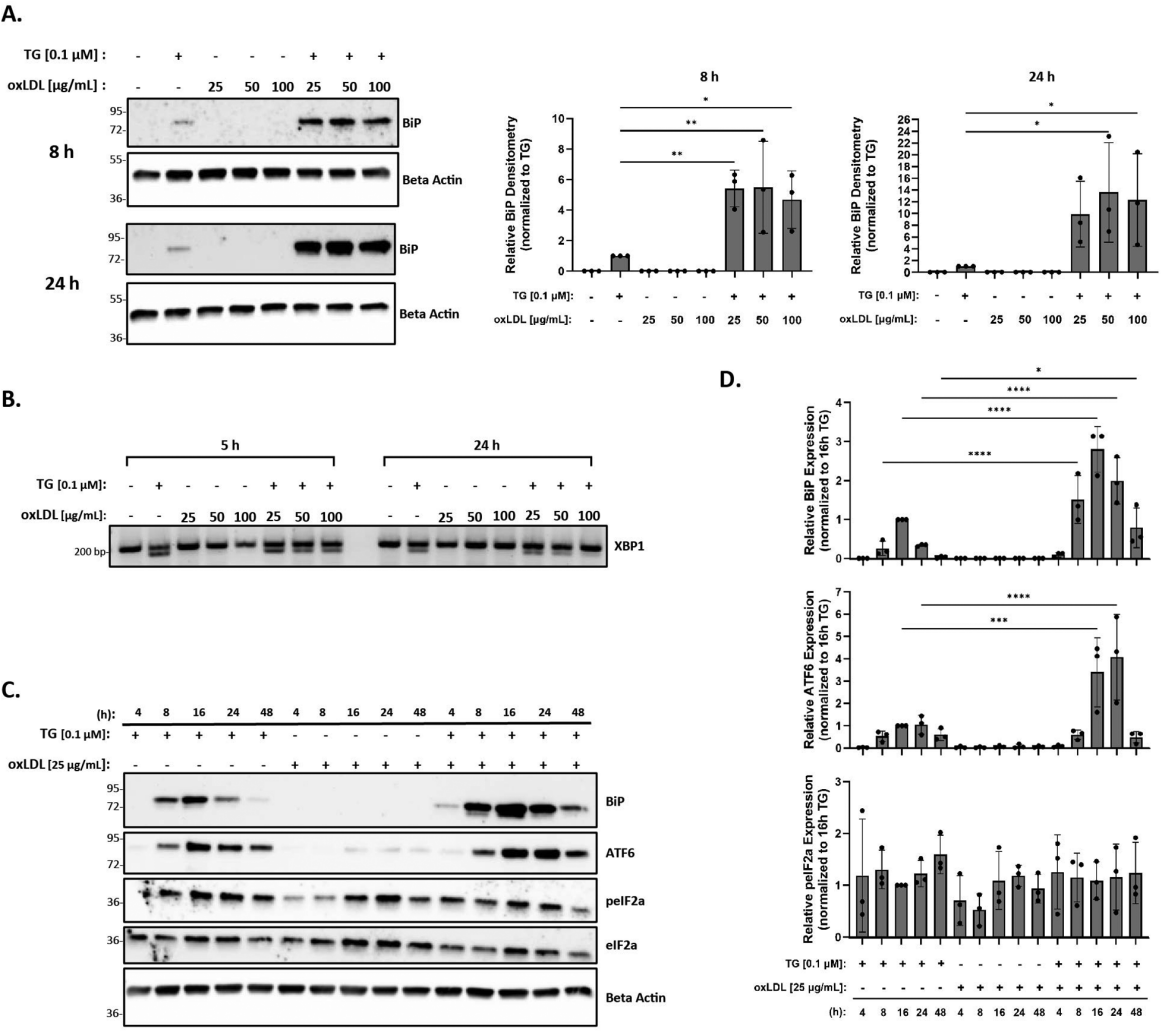
OxLDL does not induce ER stress in murine peritoneal macrophages at physiological concentrations. Murine peritoneal macrophages were treated with 25–100 µg/ml oxLDL in the presence or absence of 0.1 µM thapsigargin (TG). **(A)** Whole cell lysates were harvested and analyzed for BiP expression by immunoblot analyses. **(B)** RNA was isolated, and reverse transcribed to cDNA. Primers designed for the unspliced (200 bp) and spliced (174 bp) forms of XBP-1 were used in a semi-quantitative PCR reaction and products were separated on a 3% agarose gel. **(C)** Peritoneal macrophages were treated with 25 µg/ml oxLDL in the presence or absence of 0.1 µM thapsigargin for 4–48 h. Whole cell lysates were harvested and analyzed for expression of several ER stress markers by immunoblot analyses. **(D)** Densitometry was performed to calculate changes in band intensity. Data were first normalized to beta-actin and then normalized to thapsigargin expression at 16 h. Statistical analyses were determined by one-way ANOVA with Sidak's multiple comparison *post hoc* analysis. *p < 0.05, **p < 0.01, ***p < 0.001, ****p < 0.0001. Immunoblots and agarose gels are representative of *n* = 3 independent isolations.

The levels of oxLDL in the human body range from 6 µg/ml to 33 µg/ml, with a concentration above 15 µg/ml being common in patients with coronary artery disease (63). However, most reports demonstrating oxLDL-induced ER stress in RAW 264.7 and THP-1 macrophages (43, 52, 54, 56) used supra-physiological levels of 100 µg/ml oxLDL or above. As such, our initial goal was to demonstrate that we could reproduce ER stress in the presence of oxLDL as observed in previous studies (43, 52, 54), but at physiological concentrations. Peritoneal macrophages were treated with oxLDL ranging from 25 to 100 µg/ml (total protein concentration) for up to 24 h and UPR markers, BiP and spliced XBP-1, were analyzed to assess ER stress. We repeatedly found that treatment with oxLDL alone at all concentrations was unable to induce BiP expression (Figure 2A) or splicing of XBP-1 (Figure 2B) at all tested time points. However, we were able to demonstrate that co-incubation of macrophages with thapsigargin and oxLDL for 8 h induced BiP expression up to 5.5-fold for all concentrations of oxLDL as compared to macrophages treated with thapsigargin alone (Figure 2A). Furthermore, co-incubation of macrophages with thapsigargin and oxLDL for 24 h induced 10- to 13-fold higher levels of BiP expression as compared to thapsigargin-treated macrophages (Figure 2A). Additionally, splicing of XBP-1 was evident in macrophages co-treated with 25–100 oxLDL µg/ml and thapsigargin at all concentrations at 5 and 24 h (Figure 2B). Based on these findings, we chose to use 25 µg/ml oxLDL for subsequent experiments to keep concentrations of this lipoprotein within physiological levels.

Next, we investigated the ER stress response over a time course in macrophages treated with oxLDL, but this time, we included additional UPR markers in our assessment. In these studies, peritoneal macrophages were treated with 25 µg/ml oxLDL in the presence or absence of thapsigargin at various time points between 4 and 48 h. BiP, p-eIF2α, and ATF6 protein expression were then quantified to assess ER stress. While we did not observe ER stress in the presence of oxLDL alone at all time points, we did observe an initiation of ER stress by 8 h, a maximal ER stress response around 16 h, and a resolution of ER stress by 48 h in thapsigargin-treated macrophages (Figure 2C). Similar expression patterns of BiP and ATF6 in macrophages co-treated with oxLDL and thapsigargin were observed, with early signs of stress being evident at 4 h. Again, maximal ER stress was observed at 16 h and persisted up to 48 h (Figures 2C,D). However, no apparent pattern was observed in phosphorylated eIF2α across the various treatment groups and time course. Additional time points were also investigated for splicing of XBP-1 and no changes in response to oxLDL treatments alone were observed across time points (Supplementary Figure S1).

### 3.3 HDL does not blunt the ER stress response induced by thapsigargin in the presence or absence of oxLDL

**Figure 3:**
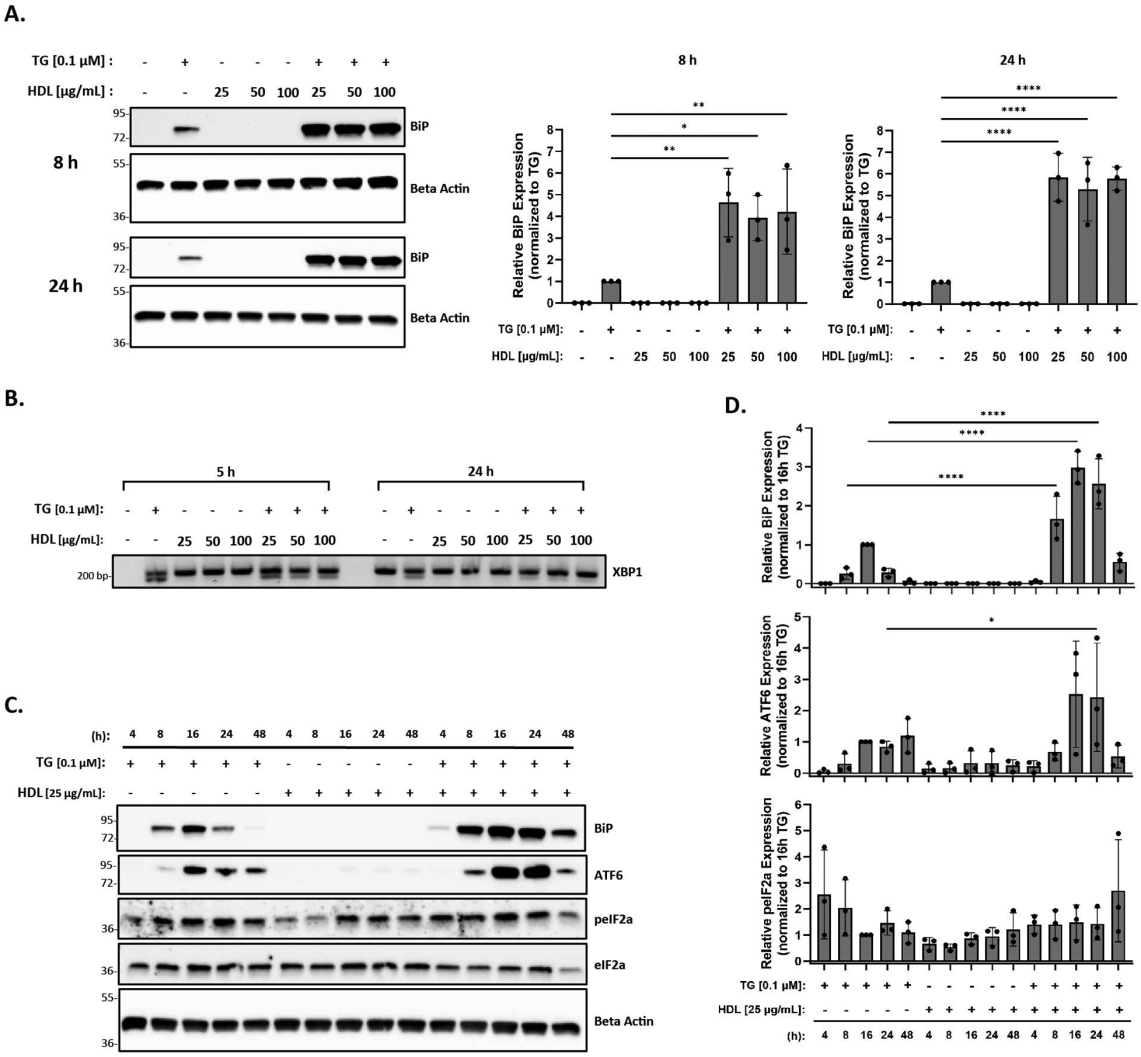
HDL does not blunt the ER stress response induced by thapsigargin. Murine peritoneal macrophages were treated with 25–100 µg/ml HDL in the presence or absence of 0.1 µM thapsigargin (TG). **(A)** Whole cell lysates were harvested and analyzed for BiP expression by immunoblot analyses. **(B)** RNA was isolated, and reverse transcribed to cDNA. Primers designed for the unspliced (200 bp) and spliced (174 bp) forms of XBP-1 were used in a semi-quantitative PCR reaction and products were separated on a 3% agarose gel. **(C)** Peritoneal macrophages were treated with 25 µg/ml HDL in the presence or absence of 0.1 µM thapsigargin for 4–48 h. Whole cell lysates were harvested and analyzed for expression of several ER stress markers by immunoblot analyses. **(D)** Densitometry was performed to calculate changes in band intensity. Data were first normalized to beta-actin and then normalized to thapsigargin expression at 16 h. Statistical analyses were determined by one-way ANOVA with Sidak's multiple comparison *post hoc* analysis. *p < 0.05, **p < 0.01, ***p < 0.001, ****p < 0.0001. All immunoblots and agarose gels are representative of *n* = 3 independent isolations.

Previous studies in vascular cells and THP-1 macrophages have shown that HDL reduces ER stress induced by oxLDL in the presence of tunicamycin (an ER stress inducer) (56). Therefore, we hypothesized that the addition of HDL to thapsigargin-treated macrophages in the presence or absence of oxLDL would similarly result in decreased ER stress. In this set of studies, peritoneal macrophages were incubated with 25–100 μg/ml HDL in the presence or absence of thapsigargin for 8 and 24 h. As expected, we found that incubation with HDL alone was unable to trigger ER stress at 8 or 24 h across concentrations (Figure 3A). Contrary to what we expected, we observed that the addition of HDL to thapsigargin-treated peritoneal macrophages resulted in a four- and five-fold increase in BiP expression at 8 and 24 h, respectively, as compared to macrophages treated with thapsigargin only (Figures 3A,D). Furthermore, co-treatment of macrophages with thapsigargin and HDL also resulted in splicing of XBP1 at 5 or 24 h (Figure 3B). Similar to what we observed with oxLDL, co-incubation of HDL with thapsigargin resulted in maximal expression of BiP and ATF6 around 16 h, with resolution of stress occurring after 48 h and variable phosphorylation of eIF2α between treatment groups and times (Figures 3C,D).

**Figure 4:**
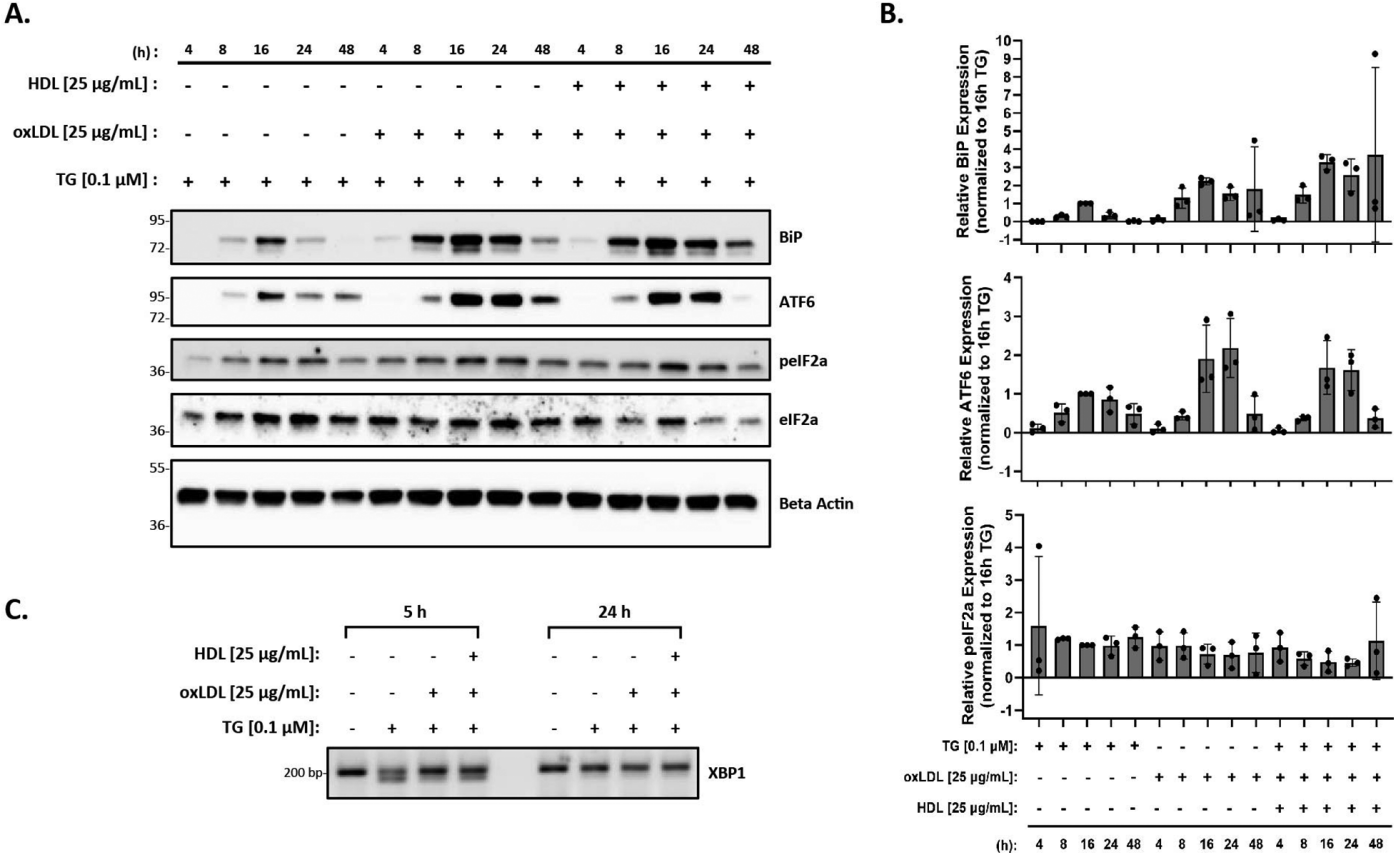
HDL does not blunt the ER stress response induced by oxLDL in the presence of thapsigargin. Murine peritoneal macrophages were treated with 0.1 µM thapsigargin (TG) in the presence or absence of 25 µg/ml oxLDL and/or HDL for indicated treatment times. **(A)** Whole cell lysates were harvested and analyzed by immunoblot analyses. **(B)** Densitometry was performed to calculate changes in band intensity. Data were first normalized to beta-actin, and then normalized to thapsigargin expression at 16 h. Statistical analyses were determined by one-way ANOVA with Sidak's multiple comparison *post hoc* analysis. *p < 0.05, **p < 0.01, ***p < 0.001, ****p < 0.0001. **(C)** RNA was isolated, and reverse transcribed to cDNA. Primers designed for the unspliced (200 bp) and spliced (174 bp) forms of XBP-1 were used in a semi-quantitative PCR reaction and products were separated on a 3% agarose gel. The immunoblots and gel shown are representative of *n* = 3 independent isolations.

Finally, as HDL has been shown to blunt oxLDL-mediated ER stress in vascular cells and THP-1 macrophages (56), we investigated the effects of HDL on macrophage ER stress induced by oxLDL in the presence of thapsigargin. Peritoneal macrophages were treated with thapsigargin and 25 µg/ml of oxLDL and HDL for various time points. Protein expression of BiP, p-eIF2α, and ATF6, along with splicing of XBP-1 were analyzed. We observed that the addition of HDL did not change the expression of BiP, p-eIF2α or ATF6 that was already induced by thapsigargin and oxLDL (Figure 4A). In fact, based on the densitometry of the bands, it appears that expression of ER stress markers may increase, although the changes are not statistically significant when all replicates are combined (Figure 4B). Similar patterns were noted for splicing of XBP1 at 5 h, however, by 24 h, only unspliced XBP1 was observed (Figure 4C).

### 3.4 Observations are independent of lipoprotein source, ER stressor, and cell type

The fact that we were unable to reproduce observations from previous studies raised several questions. First, we wanted to investigate an alternative ER stressor, tunicamycin, to use in our co-incubations. It has been demonstrated that THP-1 macrophages co-treated with oxLDL and tunicamycin had increased ER stress as compared to macrophages treated with tunicamycin; furthermore, HDL was able to reduce this ER stress (56). We tried replicating these findings and observed increased BiP expression in macrophages treated with oxLDL in the presence of tunicamycin as compared to macrophages treated with tunicamycin only (Supplementary Figure S2). However, we did not observe reduced expression of BiP in the presence of tunicamycin and HDL.

Second, we wanted to test whether the quality, source and function of lipoproteins were variables in our studies. In these experiments, we tested two batches of lipoproteins. In the first set, we examined ER stress in the presence of oxLDL and HDL that were purchased from a vendor. In the second set, HDL and LDL were isolated by density gradient ultracentrifugation (DGUC) of human plasma (58), and isolated LDL was then oxidized using the copper sulfate method [adapted from (64)]. As shown in Supplementary Figure S3A, we observed the same ER stress patterns with both sources of oxLDL, where neither batch of oxLDL was able to induce ER stress on its own. Similarly, HDL from either source was unable to blunt ER stress in response to a co-incubation of oxLDL or tunicamycin (Supplementary Figure S3B). We additionally performed an extra purification step during the isolation of HDL by DGUC by performing FPLC on the HDL fraction to ensure the removal of the Lp(a) fraction. Interestingly, this batch of HDL resulted in a greater additive effect on ER stress when incubated with thapsigargin-treated macrophages in the presence or absence of oxLDL (Supplementary Figure S3C). Therefore, the lipoprotein source was excluded as a possible variable in our studies. Furthermore, all data presented in this study relied on lipoproteins isolated by DGUC. Finally, we verified the function of our lipoproteins in primary macrophages. Specifically, we performed a cholesterol efflux assay with HDL and demonstrated that HDL could accept [^3^H]cholesterol that was effluxed from bone marrow derived macrophages (Supplementary Figure 4A). Additionally, we incubated peritoneal macrophages with oxLDL for 24 h and established that it could generate foam cells as seen by increased Oil red O staining. (Supplementary Figure S4B).

Last, we investigated ER stress in response to lipoproteins in two other cell types. We first tested bone marrow-derived macrophages as an alternate primary macrophage model. However, we observed the same trends in these macrophages such that HDL is unable to blunt oxLDL-induced ER stress in the presence of thapsigargin (Supplementary Figure S5A). We next repeated a similar experiment in human microvascular endothelial cells (HMEC-1s). Unlike what was previously reported (49), we did not observe oxLDL-induced ER stress in this cell line, nor was HDL able to blunt the ER stress response (Supplementary Figure S5B).

### 3.5 Native HDL and modified HDL exhibit similar ER stress patterns

**Figure 5:**
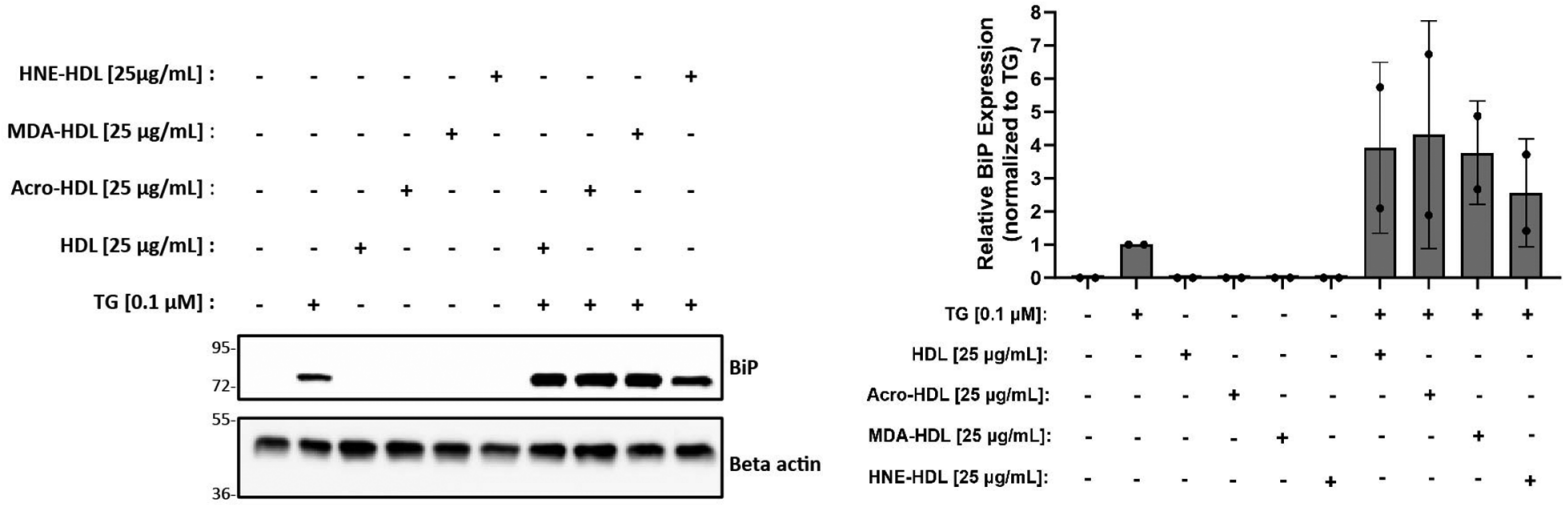
HDL modified by reactive aldehydes does not induce ER stress. Murine peritoneal macrophages were treated with 0.1 µM thapsigargin (TG) in the presence or absence of 25 µg/ml native HDL or HDL modified by acrolein, MDA, or 4-HNE for indicated treatment times. Whole cell lysates were harvested and analyzed by immunoblot analyses. Densitometry was performed to calculate changes in band intensity. Data were first normalized to beta-actin, and then normalized to thapsigargin expression. The immunoblot shown is representative of *n* = 2 independent isolations.

The initial goal of this study was to investigate whether modification of HDL by reactive aldehydes would impair HDL's ability to blunt the ER stress response in macrophages. Despite our inability to demonstrate that HDL on its own could blunt ER stress induced by thapsigargin in the presence or absence of oxLDL, we moved forward to test whether HDL modified by reactive aldehydes could increase ER stress levels. HDL was modified by acrolein, malondialdehyde (MDA) or 4-HNE and the presence of modifications was verified as previously described (27, 28, 60). In addition, TBAR or EMSA assays were performed to verify oxidation levels and changes in charge, respectively (Supplementary Figures S6). As shown in Figure 5, all three forms of modified HDL exhibited similar ER stress patterns as HDL, suggesting that the presence of reactive aldehydes does not alter HDL's role in ER stress response pathways unless in the presence of thapsigargin.

## 4 Discussion

Our initial goal of investigating modified HDL's role in mediating macrophage ER stress was challenged by our inability to replicate data that would serve as controls in our originally planned experiments. However, our observations did lead us to systematically determine the role of oxLDL and HDL in mediating ER stress responses in thioglycolate-elicited murine peritoneal macrophages. Our comprehensive studies led us to three novel discoveries in macrophages: (i) oxLDL alone was unable to induce ER stress; (ii) co-incubation with oxLDL HDL, or modified forms of HDL in the presence of thapsigargin had an additive effect on expression of ER stress markers that was prolonged as compared to cells treated with thapsigargin alone; and (iii) HDL, in the presence or absence of reactive aldehydes, was unable blunt the ER stress induced by thapsigargin in the presence or absence of oxLDL.

Our experimental conditions were modeled after numerous studies that have shown that oxLDL induces ER stress in macrophages (43, 52, 54, 56). Most studies report the use of 0.5 μM–1 μM thapsigargin to induce ER stress (61), and we questioned whether this range was too high, and possibly explained why we were unable to observe a decrease in ER stress when HDL was added to cells in some of our initial studies (data not shown). After testing a range of concentrations, we found that as little as 0.1 μM thapsigargin alone or in the presence of oxLDL was sufficient to induce ER stress in macrophages (Figures 1, 2). However, even at this lower concentration, HDL was still unable to decrease ER stress induced by oxLDL co-incubated with thapsigargin (Figure 4).

Several reports also used supra-physiological concentrations of lipoproteins in their studies that may not be suitable for cell culture treatments (63, 65). In our studies, our goal was to identify a range of lipoproteins concentrations that would be physiologically relevant and sufficient to demonstrate ER stress in the presence of thapsigargin (Figure 2). Based on our data, we chose to use 25 ug/ml for both oxLDL and HDL as it is on the lower end of physiological concentrations. Further, 25 ug/ml oxLDL is also sufficient to trigger phenotypic switching in murine primary peritoneal macrophages (66), a process that could be a trigger for the initiation of atherosclerosis.

Based on previous studies (55, 56), we hypothesized that the addition of HDL to oxLDL- and thapsigargin-treated macrophages would result in decreased ER stress. It has been proposed that HDL reduces ER stress by removing excess cholesteryl esters that were likely deposited into macrophages by oxLDL. However, we did not observe a decrease in ER stress induced by oxLDL in the presence of thapsigargin with the addition of HDL (Figure 4). Like Muller et al. (55), we tried pre-incubating macrophages with HDL prior to treatment with oxLDL in the presence or absence of thapsigargin. We also performed experiments where macrophages were first lipid-loaded with oxLDL in the presence or absence of thapsigargin for up to 24 h, prior to treatment with HDL (56). However, both methods resulted in the same observations where oxLDL did not induce ER stress and HDL could not reduce the additive ER stress induced by the co-incubation of thapsigargin and oxLDL (data not shown). Even more surprising was that HDL co-incubated with thapsigargin resulted in the same additive ER stress response as observed with oxLDL. While the mechanisms driving this synergistic effect are unknown, we speculate that oxLDL and HDL may mediate independent signaling pathways that lead to the ER stress response. Another explanation could be due to the fact that HDL particles are quite heterogeneous as shown by proteomic analyses (67), and it is possible that unique proteins within the HDL proteome that may be able to enhance the amount of ER stress when stress is already present in the cell. Further, while we layer HDL with argon to prevent oxidation, we cannot exclude the possibility that the HDL we use may harbor certain modifications (other than reactive aldehydes) that could be responsible for altering HDL's ability to blunt thapsigargin- or oxLDL-induced ER stress. Further investigations are required to better understand HDL's role in the ER stress response.

Given that HDL itself was unable to blunt ER stress, it was difficult to test our original hypothesis that modification of HDL by reactive aldehydes would prevent HDL's ER stress blunting functions. In fact, our observations suggested that HDL, whether modified by acrolein, HNE or MDA, behaved just like unmodified HDL as demonstrated by similar levels of BiP expression in the presence of thapsigargin (Figure 5). The finding that modified forms of HDL, on their own, did not induce ER stress in the macrophages was somewhat surprising as we have previously demonstrated that modified HDL increases lipid accumulation in macrophages (27), a process that has been shown to be accompanied by ER stress (50). In fact, it has also been shown that copper-modified HDL causes intracellular lipid accumulation (51, 68) in which promotes pro-atherogenic mechanisms such as ER stress-induced apoptosis (51) and inflammation (68).

The unfolded protein response is an adaptive cellular response that helps to decrease ER stress. As such, the expression of ER stress markers should start to decrease after homeostasis is restored. This made us question whether we were missing the time point at which oxLDL begins to trigger ER stress in our macrophage model. Therefore, we assessed ER stress markers at various time points ranging from 4 to 48 h. In doing so, we were able to capture time points with our positive control, thapsigargin, where we observed no ER stress markers expression (4 h), a maximal ER stress response (16–24 h), and the resolution of ER stress (48 h). Similar expression patterns in the rise and fall of ER stress markers were observed when macrophages were incubated with oxLDL or HDL in the presence of thapsigargin. Interestingly, in our hands, oxLDL on its own was unable to trigger ER stress. These observations are remarkably similar to those made by the Tabas group where oxLDL alone was unable to induce apoptosis in primary peritoneal macrophages. Rather, it was co-incubation of macrophages with oxLDL and thapsigargin that synergistically induced apoptosis (69). We speculate that oxLDL alone may not deliver enough free cholesterol into the macrophages to have the cytotoxic effects reported in Feng et al. (50) where macrophages were treated with acetylated LDL in the presence of an ACAT inhibitor. We also investigated whether treatment with acLDL alone could induce ER stress in our macrophages, however, we still found that similar to oxLDL, co-incubation with thapsigargin was needed to induce expression of BiP (Supplementary Figure S7). We speculate that the small amount of free cholesterol delivered to the cells by oxLDL (or acLDL) may result in an ER stress response in the resilient macrophage only in the presence of another source of stress in the system, such as treatment with thapsigargin or tunicamycin. Further studies are required to reveal the mechanisms by which oxLDL cooperates with thapsigargin to increase the ER stress response compared to thapsigargin treatment alone.

While our experimental conditions were similar to previously published studies, we searched for a rational explanation for why we were unable to demonstrate oxLDL-induced ER stress, as well as the blunting effect of HDL on ER stress. Towards these efforts, we took several different approaches: we tested the effects of tunicamycin as a different ER stressor, we compared different sources of lipoproteins, as well as their purity. We also tested a range of lipoprotein concentrations in our experiments. In the end, none of these factors appeared to be important variables in our assessment of ER stress. These observations led us to postulate that there could be an inherent difference in ER stress patterns when using primary macrophages as a model system as we used in our studies compared to RAW 264.7 or THP-1 cells. Very few studies have demonstrated the expression of UPR and apoptosis markers in primary macrophages in response to modified forms of LDL or HDL (50, 51, 70), although we were unsuccessful at reproducing these data. On the other hand, while THP-1 or RAW 264.7 macrophages are commonly used for their relative ease of culturing and assay flexibility, THP-1 cells are a human leukemia monocytic cell line, while RAW 264.7 cells are monocyte/macrophage-like cells that originated from a tumor in a mouse induced with the Abelson murine leukemia virus. As such, primary macrophages are considered a physiologically relevant model system for *in vitro* studies, which is an important consideration when studying specific disease mechanisms. Further investigations will be needed to distinguish differences in ER stress responses between cancer-derived macrophage lines and primary macrophages.

In conclusion, while the purpose of this study was to define the role of reactive aldehyde-modified HDL in ER stress, our experiments took an unexpected turn. Instead, using a highly systemic approach and through very convincing and reproducible data, we report alternate roles of oxLDL and HDL in mediating ER stress in thioglycolate-elicited murine peritoneal macrophages.

## 5 Conclusions

This study relied on an in-depth, systematic approach to identify the role of native and modified HDL in mediating primary macrophage ER stress. We discovered that lipoproteins on their own require the presence of thapsigargin to synergistically increase expression of ER stress markers. We further demonstrated that HDL, in the presence or absence of reactive aldehydes, was unable blunt the ER stress induced by thapsigargin in the presence or absence of oxLDL. Together, our findings suggest the need for more detailed investigations to better understand the role of native and modified lipoproteins in mediating ER stress pathways.

## Data Availability

The raw data supporting the conclusions of this article will be made available by the authors, without undue reservation.
