# Supplementary material for: A comprehensive analysis of the role of native and modified HDL in ER stress in primary macrophages
Source: Front Cardiovasc Med. 2024 Sep 12;11:1448607. doi: 10.3389/fcvm.2024.1448607 (PMC11424405; doi:10.3389/fcvm.2024.1448607)
Supplement: Supplementary file 1 [file Datasheet1.pdf]

Supplementary Materials

1. Supplementary Figures

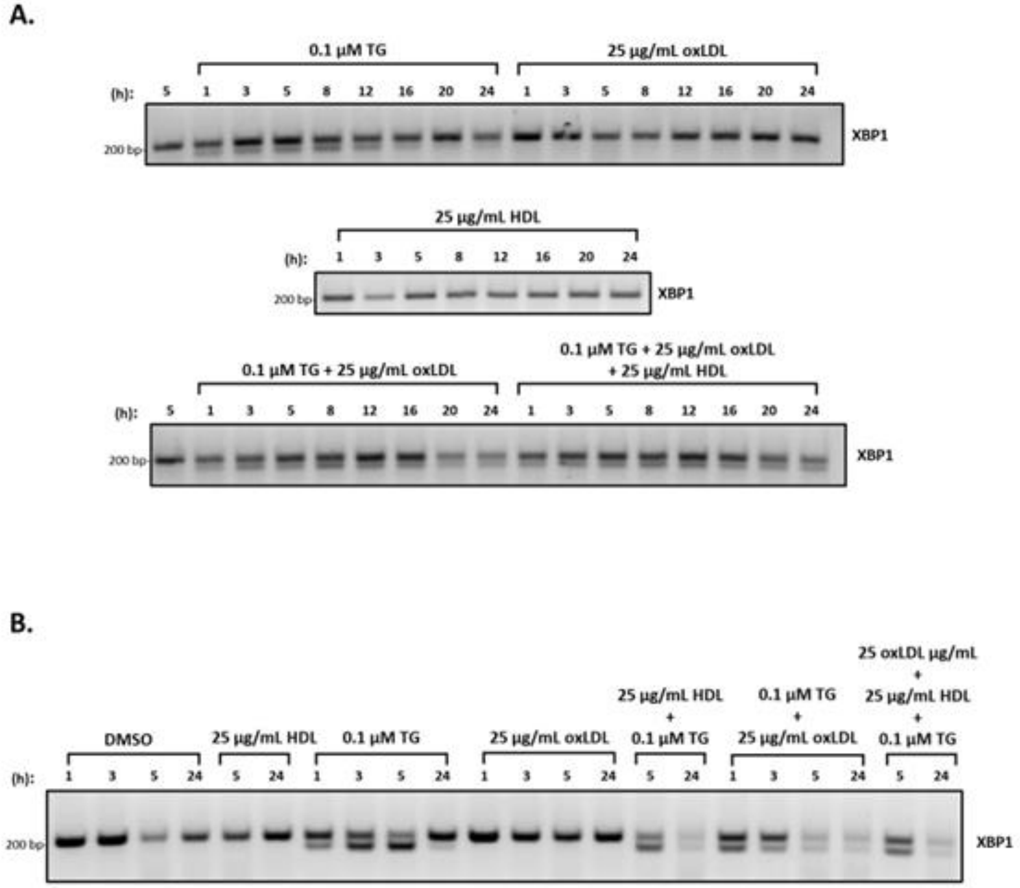

Supplemental Figure 1. HDL and oxLDL do not induce XBP-1 splicing over time. Murine peritoneal macrophages were treated with 0.1 µM thapsigargin (TG) in the presence or absence of 25 µg/mL oxLDL and/or HDL for indicated treatment times. (**A**) Whole cell lysates were harvested and analyzed by immunoblot analyses. (**B**) RNA was isolated, and reverse transcribed to cDNA. Primers designed for the unspliced (200 bp) and spliced (174 bp) forms of XBP-1 were used in a semi-quantitative PCR reaction and products were separated on a 3% agarose gel.

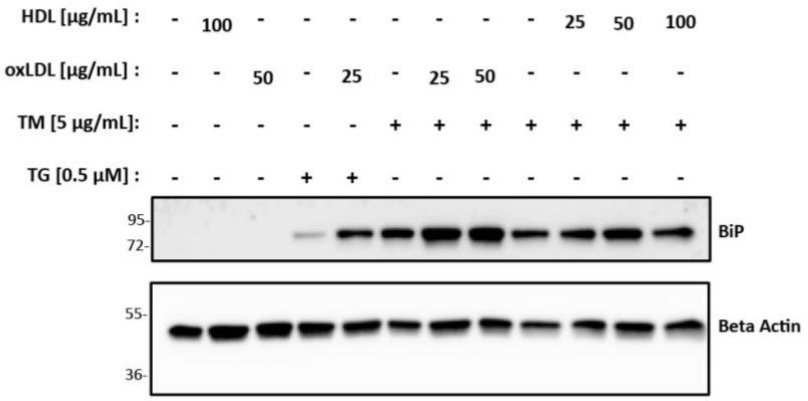

Supplemental Figure 2. Co-treatments of lipoproteins with tunicamycin revealed similar ER stress patterns. Murine peritoneal macrophages were treated with 0.5 μM thapsigargin (TG) or 5 μg/mL tunicamycin (TM) in the presence or absence of 25-100 μg/mL oxLDL and/or HDL for 8 hours. Whole cell lysates were harvested and analyzed for BiP expression by immunoblot analyses.

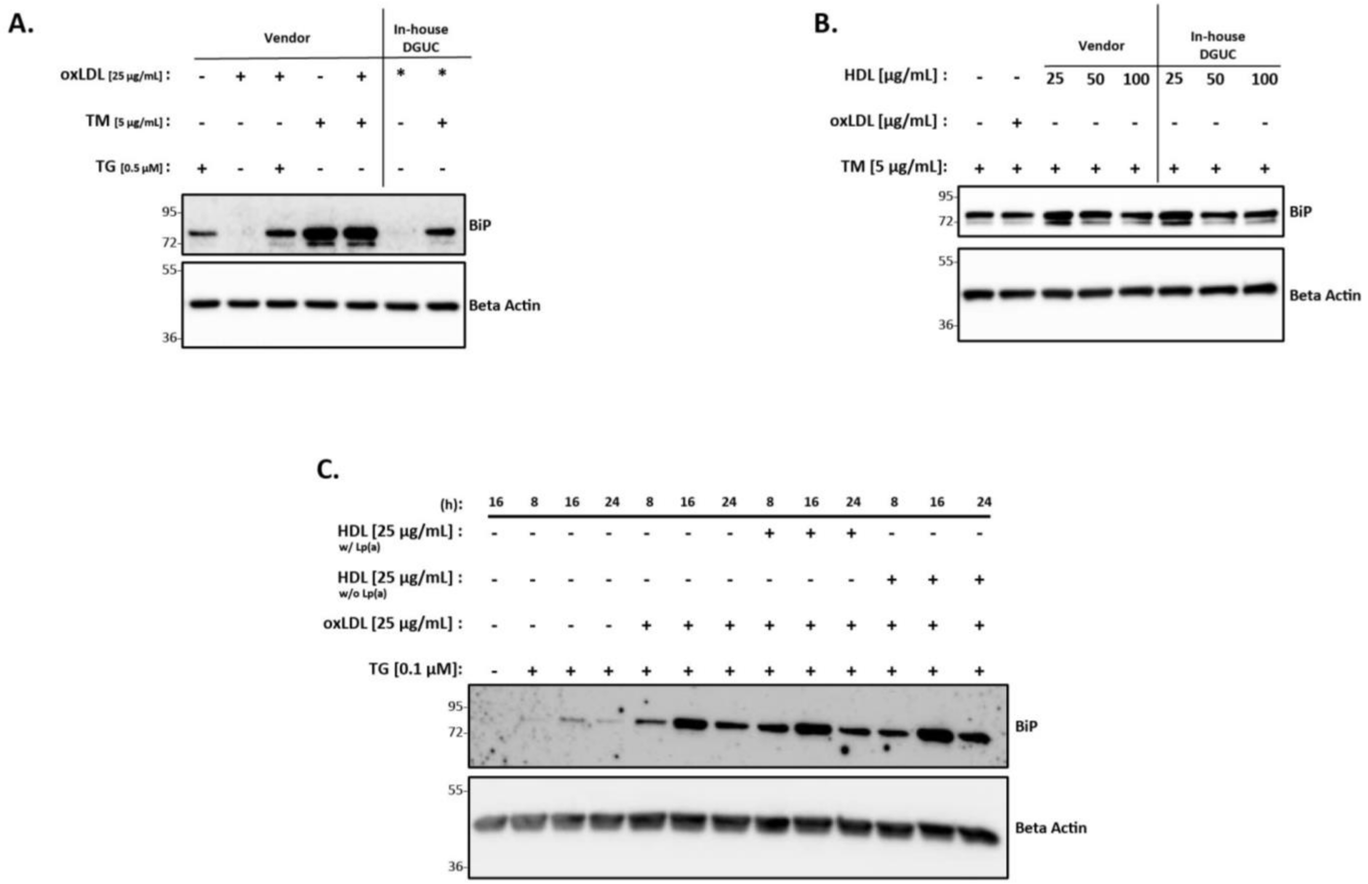

Supplemental Figure 3. Lipoprotein source or purity is not a factor in the ER stress response. (A) Murine peritoneal macrophages were treated with 0.5 µM thapsigargin (TG) or 5 µg/mL tunicamycin (TM) for 8 hours in the presence or absence of either vendor-purchased oxLDL or LDL that was isolated in house by density gradient ultracentrifugation (DGUC) and oxidized by copper for 6 hours (*).Whole cell lysates were harvested and analyzed for BiP expression by immunoblot analyses. Densitometry analyses were used to analyze changes in band intensity and were normalized to the house keeping gene, beta-actin. **(B)** Murine peritoneal macrophages were treated with 5 µg/mL tunicamycin in the presence or absence of oxLDL and/or HDL that were isolated by density gradient ultracentrifugation in house for 8 hours. Whole cell lysates were harvested and analyzed for BiP expression by immunoblot analyses. **(C)** Murine peritoneal macrophages were treated with 0.1 µM thapsigargin in the presence or absence of oxLDL and/or HDL that was either purified only by DGUC, or with HDL that was further purified by FPLC (to remove Lp(a) contamination) for indicated treatment times. Whole cell lysates were harvested and analyzed for BiP expression by immunoblot analyses.

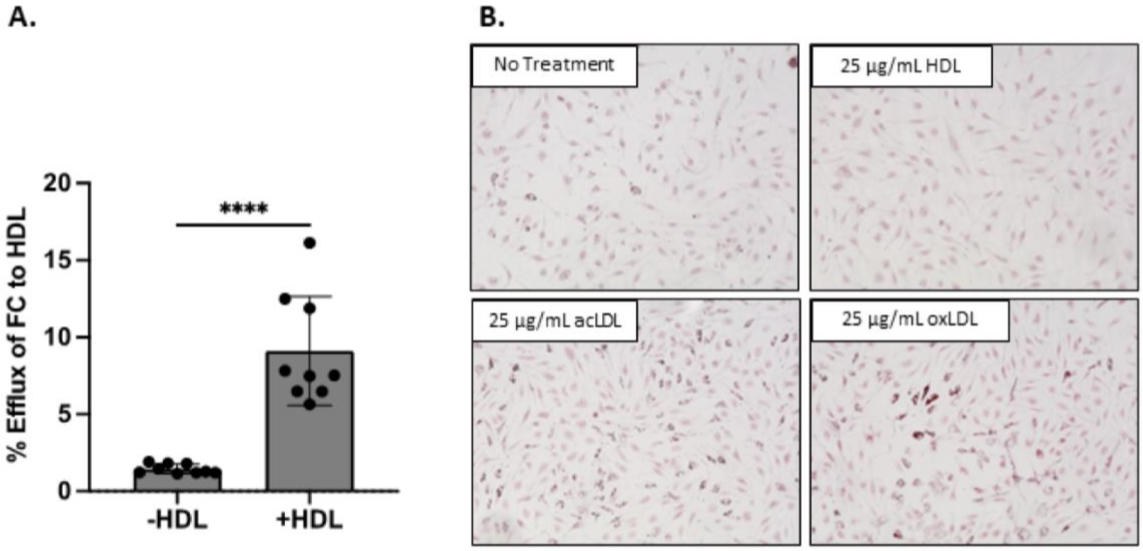

Supplemental Figure 4. HDL facilitates cholesterol efflux and oxLDL generates foam cells in macrophages. (A) Bone-marrow derived macrophages were incubated with [^{3}H]cholesterol and 50 µg/mL HDL for 4 h to test the ability of HDL to accept free cholesterol from cells. Percent efflux of free cholesterol to HDL was calculated using radioactivity counts in the cells and media. Statistical analyses were determined using unpaired T-test, $p<0.0001$. **(B)** Murine peritoneal macrophages were treated with 0.1 µM thapsigargin (TG) or 25 µg/mL oxLDL or acLDL for 24 hours. Cells were stained with Oil Red O and imaged to quantify the total amount of foam cells as compared to total cell count.

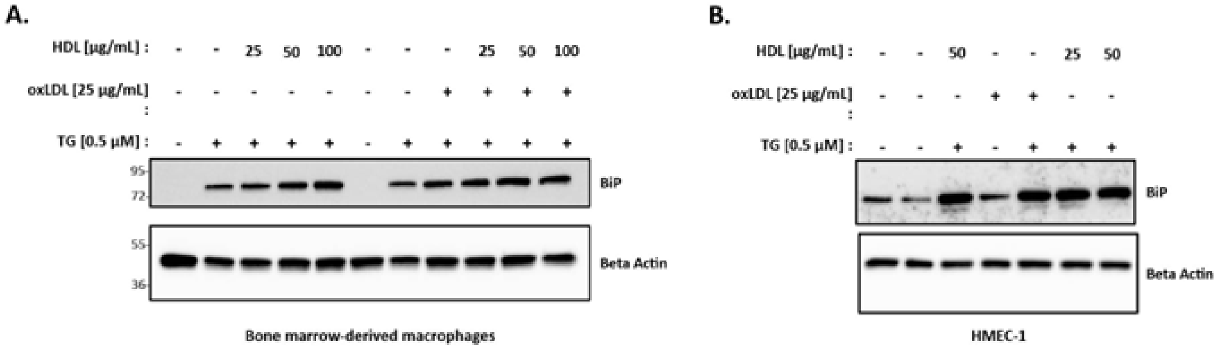

Supplemental Figure 5. HDL and oxLDL do not induce BiP expression in bone marrow-derived macrophages or cultured endothelial cells. (A) Murine bone marrow-derived macrophages or **(B)** human microvascular endothelial cells (HMEC-1s) were treated with 0.5 µM thapsigargin (TG) in the presence or absence of 25 µg/mL oxLDL and/or HDL for 8 hours. Whole cell lysates were harvested and analyzed for BiP expression by immunoblot analyses.

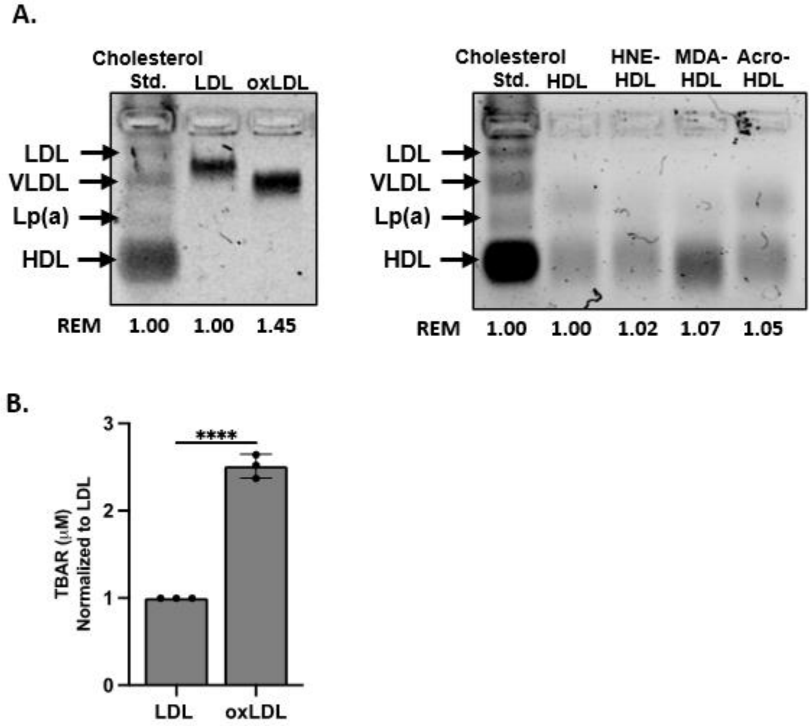

Supplemental Figure 6. Relative electrophoretic mobility shift and TBAR assays. To verify that the LDL and HDL used to generate oxLDL and modified HDL was successfully oxidized by copper sulfate and the reactive aldehydes, (**A**) agarose gels were run to determine the charge difference of the particles. **(B**) Thiobarbituric acid reactive substance (TBAR) assays were performed to verify oxidation levels of the particles. Data shown represents the mean ± SD for 3 independent oxidations. Statistical analyses were determined using unpaired T-tests, **P<0.01, and ****P<0.0001.

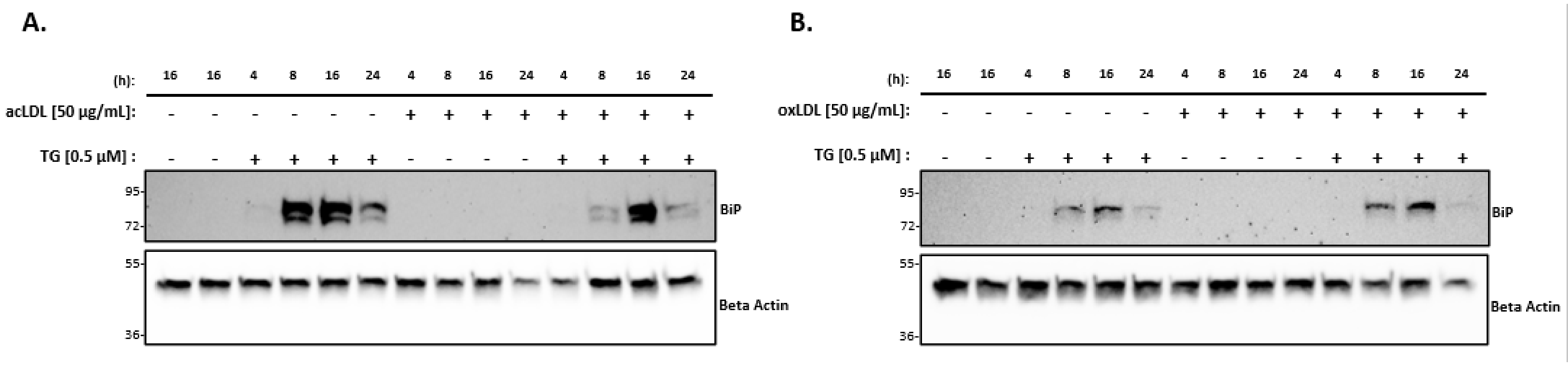

Supplemental Figure 7. acLDL and oxLDL induce similar ER stress response in peritoneal macrophages. Murine peritoneal macrophages were treated with 0.1 µM thapsigargin (TG) in the presence or absence of **(A)** 50 µg/mL acLDL or **(B)** oxLDL for up to 24 hours. Whole cell lysates were harvested and analyzed for BiP expression by immunoblot analyses.
